# Rescue Endovascular Treatment for Emergent Large Vessel Occlusion With Underlying Intracranial Atherosclerosis: Current State and Future Directions

**DOI:** 10.3389/fneur.2021.734971

**Published:** 2021-10-25

**Authors:** Sami Al Kasab, Eyad Almallouhi, Alejandro M. Spiotta

**Affiliations:** ^1^Department of Neurology, Medical University of South Carolina, Charleston, SC, United States; ^2^Department of Neurosurgery, Medical University of South Carolina, Charleston, SC, United States

**Keywords:** stroke, thrombectomy, intracranial atherosclerosis, stenting, rescue

## Abstract

Intracranial atherosclerosis (ICAS) is one of the most common causes of stroke worldwide and is associated with high risk of stroke recurrence. While the most common clinical presentation is acute–subacute transient ischemic attack or ischemic stroke, occasionally, patients with underlying ICAS present with acute occlusion of the affected vessel. Diagnosis and endovascular management of ICAS-related emergent large vessel occlusion (ELVO) can be challenging. Herein, we review the current evidence supporting endovascular management of ICAS-related ELVO and discuss future directions.

## Introduction

Endovascular treatment is the standard of care for acute ischemic stroke patients with emergent large vessel occlusion (ELVO) (1). The benefit from mechanical thrombectomy (MT) is largely driven by achieving successful revascularization (2). However, a subgroup of patients undergoing MT may have refractory occlusions due to underlying intracranial atherosclerosis (ICAS) with resultant residual severe stenosis or unstable plaque and *in situ* thrombosis/re-occlusion (3). Management of this group of patients can be challenging and commonly requires rescue therapy with intra-arterial antiplatelet, thrombolytics, anticoagulation, angioplasty, stenting, or combination of treatment modalities. The safety and efficacy of such rescue treatments have not yet been established, and current evidence is largely driven from small retrospective case series (4–6). In addition, the current literature on the prevalence and endovascular treatment of ICAS-related ELVO mostly comprised Asian studies. It is known that ICAS is more prevalent in Asian population; studies evaluating outcomes of angioplasty and/or stenting in Asian population showed promising results; on the other hand, strong evidence form randomized controlled trials in North America and Europe showed clear advantage of medical management over angioplasty/stenting (7–9). In this review, we discuss the prevalence, pathophysiology, and the available evidence supporting management of ICAS-related ELVO and provide insights on future directions.

## Prevalence, Pathophysiology, and Diagnosis

The prevalence of ICAS-related ELVO ranges from 1.9 to 30% depending on few factors including definition used, the study population included, and the involved vascular territory in the published literature (10–12). ICAS is much less common in Western population compared with Asian population (12, 13). In Asian population, ICAS-related ELVO is estimated to be responsible for approximately one-third of ELVOs (9, 13); conversely, ICAS-related ELVO is much less common in Western population and is responsible for <10% of all ELVOs (14). This likely reflects the fact that ICAS is more common in Asian than Western population (15). Previous studies evaluating risk factors for ICAS-related ELVO showed that compared with embolic ELVO, ICAS-related ELVO patients are younger and more likely to be men. In addition, patients with ICAS-related ELVO have higher incidence of hypertension, hyperlipidemia, and diabetes mellitus and lower incidence of atrial fibrillation (4). While the abovementioned risk factors are suggestive of ICAS, diagnosis of ICAS-related ELVO poses a major challenge.

The mechanism of acute/subacute strokes with ICAS had been extensively studied before (16–19). The main mechanisms responsible for TIA/stroke with ICAS are as follows: (a) *in situ* thrombosis, (b) branch atheromatous disease, (c) hemodynamic compromise, (d) artery-to-artery embolism, or (e) combination of the mechanisms (20, 21). However, for ICAS-related ELVO, the main proposed mechanism is ruptured, unstable plaque with subsequent *in situ* thrombosis and resultant occlusion and re-occlusion (22, 23).

Evaluating pre-thrombectomy imaging can be helpful in identifying patients with ICAS. Findings such as calcification of the internal carotid artery on computed tomography, clot burden on computed tomography angiography (CTA), and magnetic resonance imaging could be suggestive of ICAS as the underlying mechanism of ELVO given that ICAS patients have lower clot burden (24, 25). The finding of calcifications on baseline CT imaging particularly in the posterior circulation is indicative of ICAS (26). Previous studies have also suggested the use of baseline infarct core volume to predict ICAS. Due to better collateral circulation in patients with ICAS-related ELVO compared with embolic ELVO (13), patients with ICAS are expected to have lower infarct volume. Baseline infarct volume was assessed in one study of patients with embolic ELVO and ICAS ELVO. Patients with ICAS ELVO had significantly lower baseline infarct volume (14 vs. 54 ml in ICAS vs. embolic ELVO, respectively, *p* ≤ 0.001) (24).

The importance of collateral status in (a) aiding with the diagnosis of ICAS and (b) determining infarct volume has been studied before. A study by Lee et al. assessed leptomeningeal collateral status using the American Society of Interventional and Therapeutic Neuroradiology/Society of Interventional Radiology (ASITN/SIR) collateral grading scale (27) in patients with middle cerebral artery (MCA) M1 segment occlusion. In this study, 8/10 (80%) patients with ICAS-related ELVO had excellent collateral grade compared with 19/43 (44.2%) of patients with embolic ELVO (*p* = 0.032) (13). A recent study by Baek et al. investigated the utility of pre-procedural leptomeningeal collateral status in predicting ICAS-related ELVO (28). The authors used CTA to assess collateral status utilizing Tan's collateral grading system (29). In brief, Tan's system has four grades, from A indicating absent collateral supply to D indicating complete collateral supply of 100% of the MCA territory (29). In the study by Baek et al. 40 patients with ICAS-related ELVO and 186 with embolic ELVO were included. Complete (100%) collateral status was significantly higher among patients with ICAS-related ELVO (52.5% vs. 20.4%) (28).

**Figure 1:**
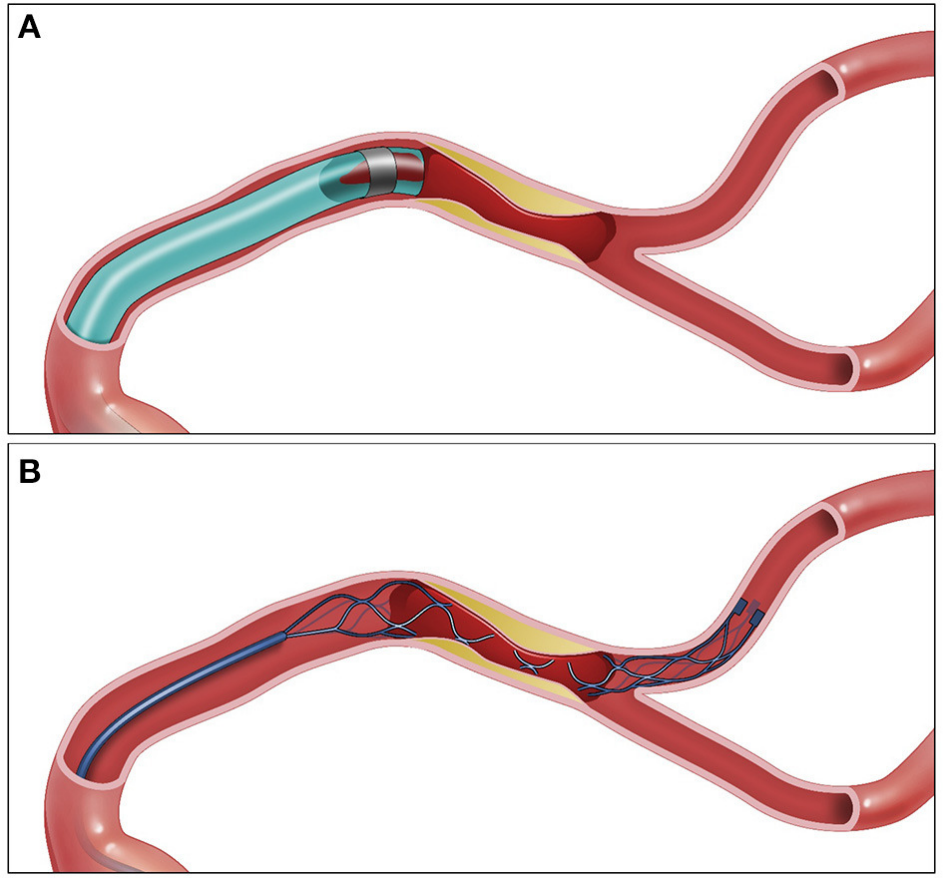
ADAPT vs. SR for EVT in ICAS ELVO. **(A)** Aspiration catheter engulfing the proximal end of the thrombus. **(B)** Stent retriever engulfing the thrombus—the stent retriever shape is changed at the location of the plaque. SR, stent retriever; ADAPT, a direct aspiration first pass technique; MT, mechanical thrombectomy; ICAS, intracranial atherosclerosis; ELVO, emergent large vessel occlusion.

**Figure 2:**
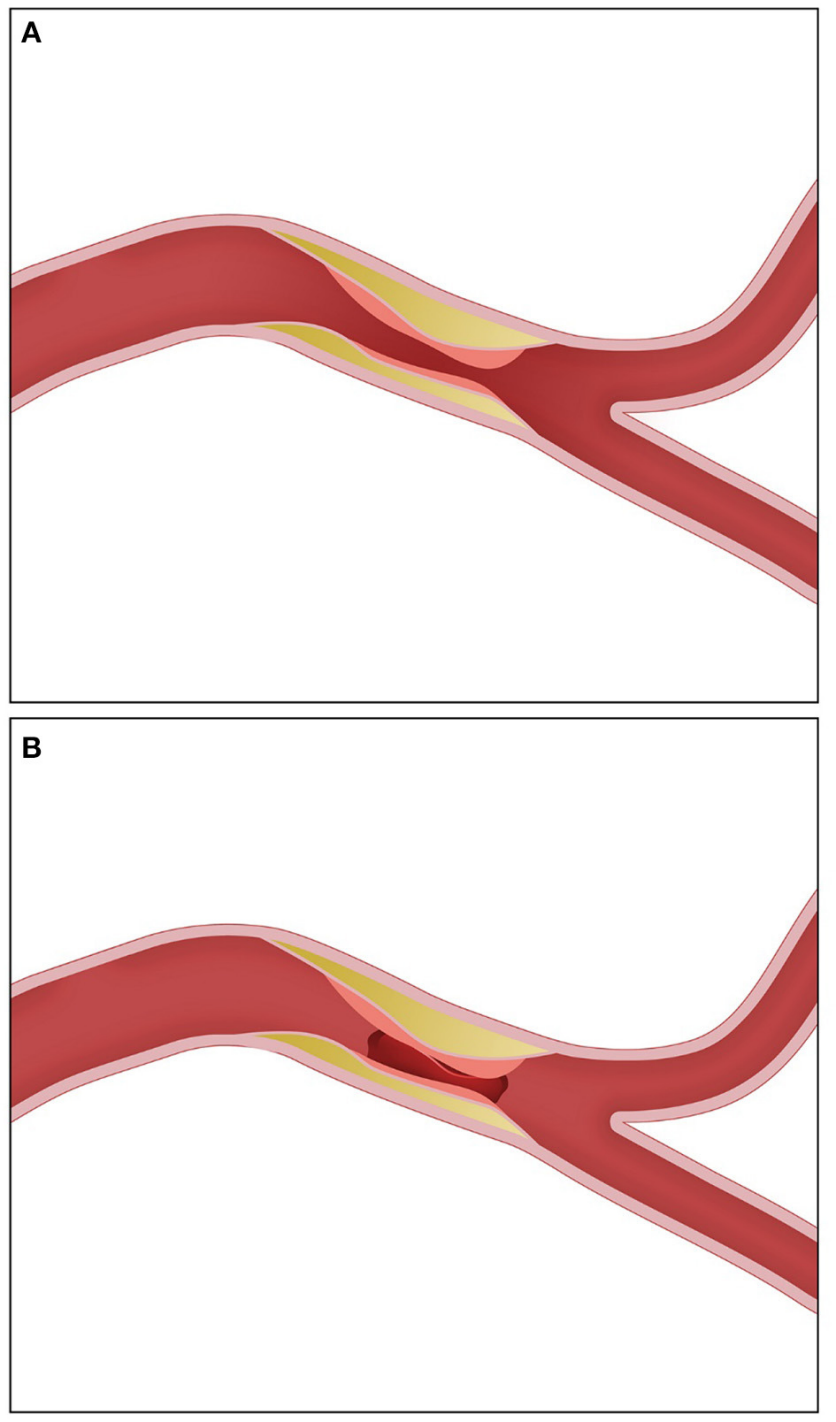
Post-thrombectomy angiographic appearance in ICAS-related ELVO requiring rescue therapy. **(A)** Severe residual stenosis. **(B)** Re-occlusion. ICAS, intracranial atherosclerosis; ELVO, emergent large vessel occlusion.

Intra-procedurally, diagnosis of ICAS as the underlying mechanism of ELVO is challenging and often times is made only following initial revascularization attempt. Occasionally, the use of stent retriever (SR) could help establish the diagnosis given that SRs often show full proximal and distal deployments but partial deployment across the stenotic area (truncal deployment) (Figure 1). In addition, the presence of robust leptomeningeal collateral on initial angiography is more likely to be found with ICAS compared with embolic ELVO (13). Nevertheless, there are currently no universally accepted diagnostic criteria; however, one study evaluated the inter-rater reliability of using the presence of any of the following: (a) residual fixed stenosis following MT that measures >50% or (b) mild–moderate stenosis with re-occlusion on follow-up angiography or (c) evidence of distal hypoperfusion and ruling out of other pathologies such as dissection (Figure 2). These criteria were validated in a previous study showing acceptable inter-rater reliability (9). While the presence of residual fixed severe stenosis following MT is the most straightforward diagnosis of ICAS, the presence of re-occlusion without associated significant stenosis can arguably be secondary to an organized partially removed thrombus. The presence of re-occlusion, however, is much less common in embolic ELVO compared with ICAS-related ELVO. A number of studies evaluated the rate of intra-procedural re-occlusion in ICAS-related ELVO compared with embolic ELVO. The rate of intraprocedural re-occlusion had been shown to be significantly higher in ICAS-related ELVO; most studies have reported a rate of 30% for ICAS-related ELVO compared with <3% for embolic ELVO (4, 30). A study by Kang et al. evaluated the rate of instant re-occlusion during MT; in their study, 40/132 (30%) had *in situ* thrombosis with re-occlusion; and all of those patients had underlying ICAS on follow-up imaging (31). The underlying mechanism of re-occlusion is not well studied. It is postulated that an underlying unstable plaque resulting in platelet activation followed by *in situ* thrombosis could result in re-occlusion. A postmortem study suggested underlying fibrous cap disruption and sub-intimal dissections as possible explanation of *in situ* thrombosis and resultant re-occlusion (30–32). Some interventionists repeat angiography 5–10 min after successful revascularization but with residual mild–moderate stenosis. Repeat angiography helps in evaluating for re-occlusion and ruling out other etiologies such as vasospasm.

## Discussion

ICAS-related ELVO poses a major therapeutic challenge; the main challenge is the tendency of re-occlusion of the affected vessel, or residual stable fixed severe stenosis impairing distal flow. The optimal treatment strategy for such cases is unclear; however, it often involves the use of angioplasty and/or stenting to achieve successful revascularization. When assessing the safety and efficacy of angioplasty and stenting for ICAS, it is important to distinguish this group of patients from patients with ICAS presenting with transient ischemic attack or subacute stroke. In the latter, there is strong evidence that medical management is superior to endovascular treatment with angioplasty and/or stenting; this is derived from the peri-procedural complications related to angioplasty and advancement in medical management (33, 34). In the ICAS-related ELVO group, however, patients are at risk of suffering from a large, often disabling stroke if left with unsuccessful revascularization. Treatment options depend on the clinical scenario, mainly related to the angiographic appearance following recanalization and whether there is re-occlusion of the vessel vs. residual severe flow-limiting stenosis. Treatment options could include intra-arterial treatment with thrombolytics, antiplatelets, angioplasty, and/or stenting. The evidence of the abovementioned treatment options and approaches is not strong and derived largely from small retrospective case series. Importantly, the vast majority of these studies were carried out in Asia with only few studies coming from the United States and Europe. Aside from ICAS being more common in Asian patient population, similar to ICAS in the non-emergent setting, studies coming from the United States and Europe have shown different results as compared with those coming from Asia (5, 6, 35). While it remains unclear why there are such stark differences in treatment effects between Asian and Western patient populations with ICAS, studies have suggested genetic, socioeconomic, and dietary differences as contributing factors (14, 20). A study by Yoon et al. evaluated the safety of emergent angioplasty with or without stenting in patients with ICAS-related ELVO; the authors reported more favorable outcomes in the ICAS group compared with the control group, with no difference in symptomatic intracranial hemorrhage or mortality (36). Conversely, a study from Spain by Matias-Guiu reported longer procedural times; higher mortality and lower rates of good functional outcomes in ICAS-related ELVO require rescue therapy with angioplasty with or without stenting (37). This study, however, only had 15 patients with ICAS (10, 11). Other studies reporting on comparably small sample sizes carried out in the United States and Europe have reported conflicting results with regard to rates of successful revascularization, complication rates, and long-term outcomes (4). The small sample size in those studies is likely due to the fact that ICAS is less prevalent in Western compared with Asian population (38).

In addition, the thrombectomy approach and whether an SR vs. a direct aspiration first pass technique (ADAPT) is used initially may influence the final rescue therapy treatment decision. There are advantages to each treatment approach. Although current evidence supports similar efficacy and safety profile for SR and ADAPT as frontline MT techniques, the vast majority of patients included in these trials were embolic ELVOs (39, 40). Therefore, it remains unclear what the best firstline MT treatment for ICAS-related ELVO. Advantages of SR frontline thrombectomy approach include the following: (A) Early diagnosis. Given that SR takes the shape of the stenotic area (truncal occlusion), SR could help identify the lesion following the deployment of SR (Figure 1) and therefore earlier treatment planning. (B) With SR deployment, there is partial flow restoration, which could in theory reduce clot burden. (C) Some interventionists advocate for SR as frontline approach for ICAS-related lesions due to its ability to completely engulf the clot, whereas with aspiration, the aspiration catheter tip could be against the plaque; however, with newer larger caliber aspiration catheters where there is full engagement of the lesions, this argument is unlikely to be valid (Figure 1B). The main advantages of using ADAPT as firstline treatment for ICAS-related ELVO include (a) less interaction with the plaque as opposed to SR, which could lead to further endothelial damage, plaque inflammation, and clot propagation (41, 42). Furthermore, in perforator-rich segments such as M1 segment of the MCA, and midsegment of the basilar artery, the use of SR could lead to perforator occlusion “snow plowing,” which is less likely to happen with ADAPT (43). Nevertheless, to our knowledge, there is no study to date that compared SR vs. ADAPT as frontline approach for patients with ICAS-related ELVO.

**Figure 3:**
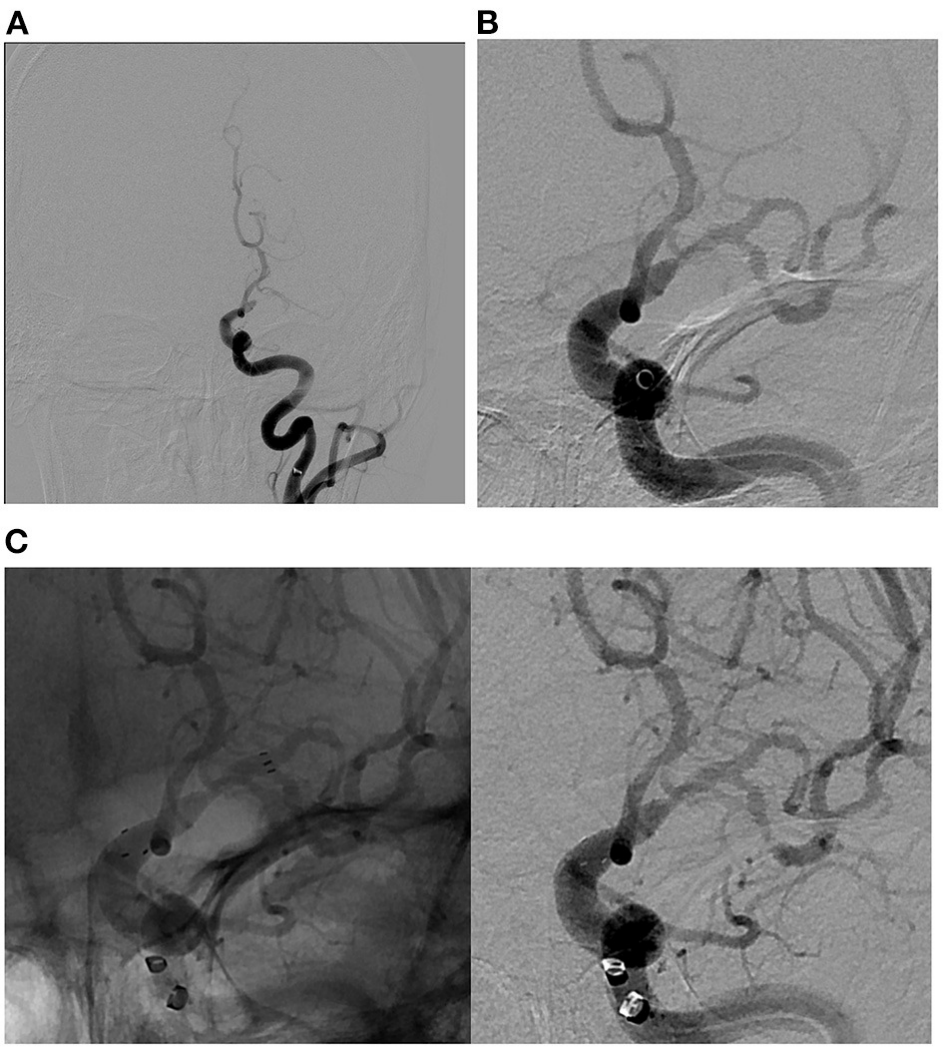
Illustrative case of ICAS-related LVO in the M1 segment of the left middle cerebral artery. **(A)** Pre-MT angiographic image demonstrating complete occlusion of M1 segment of the left middle cerebral artery. **(B)** Post-MT angiographic image demonstrating partial recanalization of M1 segment of the left middle cerebral artery with persistent severe flow-limiting stenosis. Of note, ADAPT technique was used in this case. **(C)** Native (left) and digitally subtracted (right) angiographic images demonstrating successful intracranial stenting with Neuro-Form atlas stent as a rescue therapy with a goal of maintaining cerebral flow through the stenotic portion of M1 segment of the left middle cerebral artery. ADAPT, direct aspiration first pass technique; MT, mechanical thrombectomy; ICAS, intracranial atherosclerosis; ELVO, Emergent large vessel occlusion.

**Figure 4:**
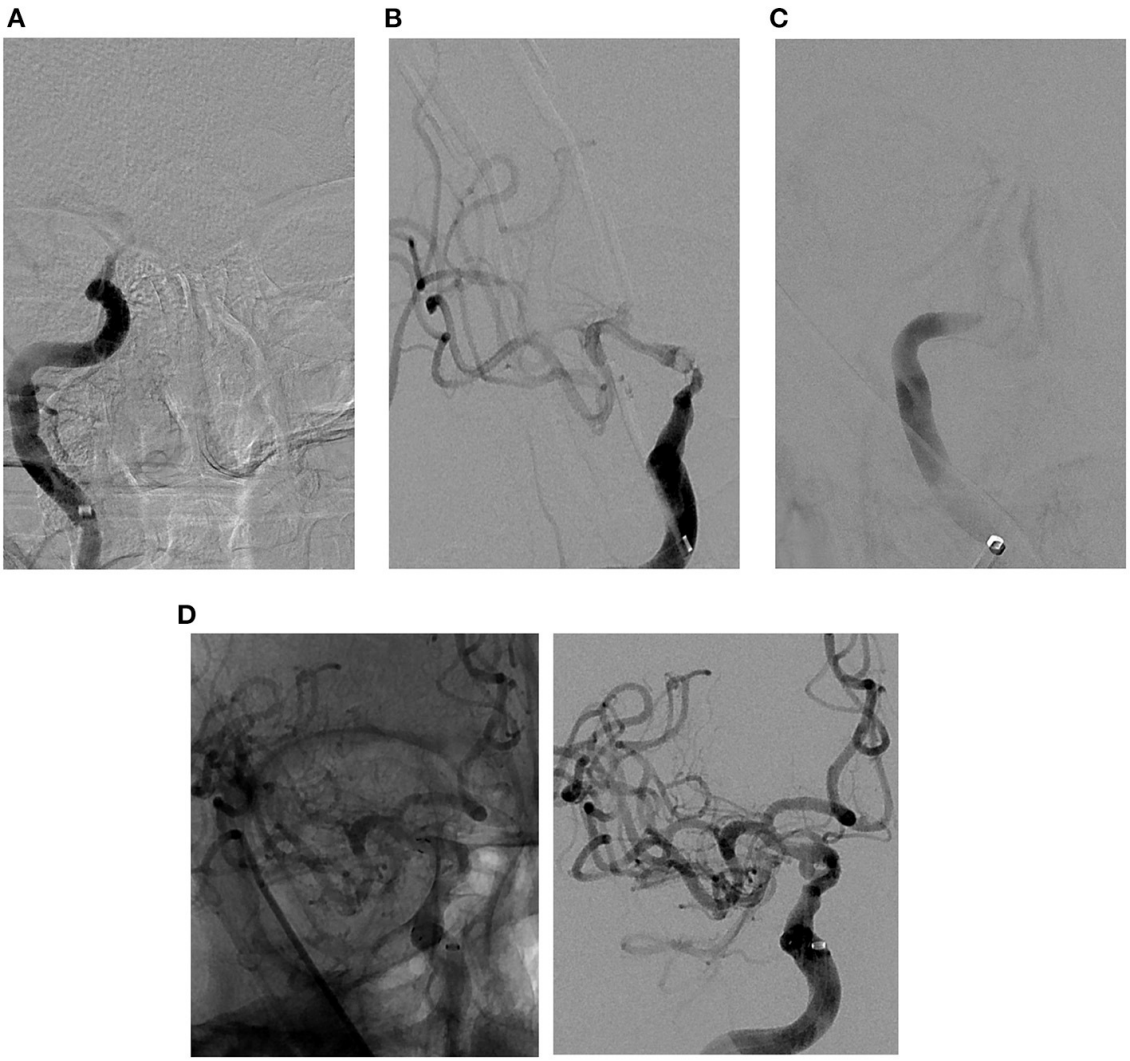
Illustrative case of ICAS-related LVO in the cavernous segment of the right internal carotid artery. **(A)** Pre-MT angiographic image demonstrating complete occlusion of cavernous segment of the right internal carotid artery. **(B)** Post-MT angiographic image demonstrating partial recanalization of cavernous segment of the right internal carotid artery with persistent severe stenosis. **(C)** Delayed angiographic image 5 min post-MT demonstrating re-occlusion of the intracranial internal carotid artery. **(D)** Native (left) and digitally subtracted (right) angiographic images demonstrating successful angioplasty and stenting as a rescue therapy, with a significant improvement in the severe stenosis and near complete recanalization of the right internal carotid artery. ADAPT, a direct aspiration first pass technique; MT, mechanical thrombectomy; ICAS, intracranial atherosclerosis; ELVO, emergent large vessel occlusion.

The approach to rescue therapy used depends on underlying lesion visualized following first successful thrombectomy pass, baseline core infarct, and involved segment. Once ICAS is identified, our practice is to repeat after waiting for 5–10 min and re-evaluate with follow-up angiography. In cases of residual severe stenosis without evidence of re-occlusion, angioplasty is performed first if lesion is not involving perforator-rich segment; if residual flow-limiting stenosis persists, stenting is then performed. In perforator-rich segments, we typically stent without balloon angioplasty (Figure 3). We avoid angioplasty in perforator-rich segments such as M1 and midbasilar segments, given associated risk of perforator occlusion. Angioplasty is typically performed using non-compliant balloon sized to the diameter of the normal intracranial segment proximal to the affected segment. Our practice has been to perform sub-maximal angioplasty to avoid perforator occlusion and reduce the amount of plaque disruption. A meta-analysis by Seyedaadat et al. evaluated 19 studies with 777 patients who underwent submaximal angioplasty for ICAS. The authors found 93% success rate with 3% stroke rate at 1 month (44), significantly lower rates than what were reported in SAMMPRIS (33) and VISSIT (5). In cases of re-occlusion, our practice has been to repeat MT and give a weight-based intra-arterial dose of tirofiban followed by stenting. Pre-stenting balloon angioplasty is performed in cases of associated severe flow-limiting stenosis (Figure 4).

The type of stent used in ICAS-related ELVO is also controversial. The ideal stent is one that is easy to deliver and has excellent radial force and low metal–vessel ratio. The Neuro-Form atlas stent is a self-expanding nitinol stent with hybrid cell structure. The stent has 6–12% vessel coverage and has a moderate radial force that is higher than flow diverters but lower than Wingspan stent (Stryker Neurovascular, Fremont, CA, USA). In our practice, we use Neuro-Form Atlas for ICAS-related ELVO with good success rate (45). In addition, balloon-mounted stents have recently emerged as potential effective stents in this population. The advantage of balloon-mounted stents is the added radial force as compared in Neuro-Form atlas (Stryker Neurovascular) and the pre-balloon angioplasty associated with deployment; however, due to their stiffness, navigating such stents could be challenging, particularly in tortuous anatomy. Mohammaden et al. recently evaluated the use of balloon-mounted stent in the treatment of symptomatic ICAS (46). Among 232 patients with symptomatic ICAS patients who failed medical management and were treated with balloon-mounted stents, 5.6% had strokes within 72 h, 3.9% were ischemic, and 1.7% were hemorrhagic. Recurrent strokes were reported in 3.7% at follow-up. While this patient population is different than ICAS-related ELVO patients, the safety profile of balloon-mounted stents in ICAS patients provides helpful data that could be used for future studies.

The use of intra-arterial thrombolytics such as alteplase (tPA) or urokinase has been suggested. However, given prior evidence of increased risk for hemorrhage, prior failure of intra-arterial tPA trials' use of intra-arterial thrombolytics is generally avoided (1, 47). One study evaluated clot composition in 37 patients with large vessel occlusion undergoing MT. In this study, patients with large artery atherosclerosis were found to have higher proportion of platelets and fibrin and lower proportion of red blood cells as compared with those with cardio-embolic occlusion (23). Given the platelet/fibrin-rich component of ICAS-related clots, the use of antiplatelet as first-line rescue therapy has been evaluated. Tirofiban is a short-acting glycoprotein IIb/IIIa inhibitor and competitively inhibits platelet aggregation mediated by fibrinogen. The use of low-dose tirofiban was evaluated on patients undergoing MT with second-generation SR evaluated in a prospective observational study in China. In this study, the use of low-dose tirofiban was not associated with increased risk of hemorrhage or long-term mortality (48). A recent study carried out in Korea compared the outcomes of using intra-arterial glycoprotein IIb/IIIa inhibitor infusion with those emergent angioplasty and found that both techniques resulted in a successful revascularization rate of about 95% with similar rates of symptomatic hemorrhage, 3-month functional independence, and mortality (49). However, one major disadvantage of intra-arterial tirofiban is late re-occlusion as reported in prior studies (31). Finally, the mechanism of stroke and location of lesion should take into account the approach to treatment, and response to intra-arterial antiplatelets, angioplasty, and/or stenting should be taken into account when attempting to identify the ideal treatment approach for ICAS-related ELVO (50).

## Future Directions

ICAS-related ELVO remains a challenging and poorly understood entity. Current evidence shows good safety and efficacy outcomes of rescue treatments with balloon angioplasty and/or stenting; however, evidence is limited to retrospective studies. Future direction should focus on attempting to identify this group of patients pre-procedurally utilizing calcium burden, collateral status, location of occlusion, and stroke severity on arrival. In addition, while there is good evidence to support the use of balloon angioplasty and/or stenting, the ideal balloon and stent used are unknown and often dependent on the interventionist preference.

Furthermore, the amount of balloon angioplasty to be performed should also be better studied. Similar to refractory ICAS in the subacute setting, submaximal angioplasty could offer similar results to maximal angioplasty with lower complication rates. Finally, the long-term impact of angioplasty and/or stenting as well as intra-arterial treatment should be studied. Future studies should focus on assessing vessel patency on follow-up imaging.

## Data Availability Statement

The original contributions presented in the study are included in the article/supplementary material, further inquiries can be directed to the corresponding author/s.

## Author Contributions

All authors listed have made a substantial, direct and intellectual contribution to the work, and approved it for publication.

## Conflict of Interest

The authors declare that the research was conducted in the absence of any commercial or financial relationships that could be construed as a potential conflict of interest.

## Publisher's Note

All claims expressed in this article are solely those of the authors and do not necessarily represent those of their affiliated organizations, or those of the publisher, the editors and the reviewers. Any product that may be evaluated in this article, or claim that may be made by its manufacturer, is not guaranteed or endorsed by the publisher.
